# D-enantiomeric antibiofilm peptides effective against anaerobic *Cutibacterium acnes* biofilm

**DOI:** 10.1128/spectrum.02523-24

**Published:** 2025-03-25

**Authors:** Jennifer Varin-Simon, Evan F. Haney, Marius Colin, Frédéric Velard, Sophie C. Gangloff, Robert E. W. Hancock, Fany Reffuveille

**Affiliations:** 1 Université de Reims Champagne-Ardenne, BIOS, Reims, Grand Est, France; 2 Université de Reims Champagne-Ardenne, UFR Pharmacie, Reims, Grand Est, France; 3 Asep Medical Inc./ABT Innovations Inc., Victoria, British Columbia, Canada; 4 Department of Microbiology and Immunology, Center for Microbial Diseases and Immunity Research, University of British Columbia 198130 , Vancouver, British Columbia, Canada; Pontificia Universidade, Curitiba, Brazil

**Keywords:** *Cutibacterium acnes*, host defense peptide, prosthesis joint injection, biofilm

## Abstract

The emergence of antibiotic resistance, biofilm formation, and internalization by host cells contribute to a high risk of chronic infections, highlighting the necessity to develop novel therapeutic strategies. Identification of natural host defense peptides (HDPs) with promising antimicrobial and antibiofilm activities led to the development of synthetic peptides with broad-spectrum efficacy. However, few studies have examined their effect on anaerobic bacterial species. This study aimed to test the effect of synthetic HDPs on *Cutibacterium acnes*, an anaerobe species involved in 10% of prosthesis joint infections (PJI). A preliminary screen identified three peptides (DJK5, AB009-D, and AB101-D) with promising activity against four *C. acnes* strains (two of which were isolated from PJI). A bactericidal effect was observed for the three peptides with 50% of planktonic bacteria killing for AB009-D and AB101-D after only 3 hours of contact. DJK5 and AB009-D inhibited the *C. acnes* adhesion on plastic and titanium supports with a 2-log decrease in bacterial cells. In the presence of peptides, the morphology of *C. acnes* cells was altered with an increase in cell length observed, especially for one of the non-PJI-related strains. Against mature biofilms, AB101-D was the most effective with an approximate 2-log decrease in adhered CFUs, indicating the induction of bacterial dispersion or death. DJK5 also inhibited *C. acnes* internalization by osteoblasts, with a reduction of the internalized bacteria quantity for three strains. Overall, this study demonstrates that synthetic HDPs are effective against anaerobic bacteria and hold promise as novel therapeutic candidates to prevent or treat *C. acnes* PJIs.

**IMPORTANCE:**

The emergence of antibiotic tolerance highlights the necessity to develop novel therapeutic strategies with promising antimicrobial but also antibiofilm activities. In this study, we tested the effect of synthetic host defense peptides (HDPs) on *Cutibacterium acnes*, an anaerobic species, rarely studied, whereas involved in 10% of prosthesis joint infections (PJI). In our study, we demonstrate that the selected synthetic HDPs are effective against this anaerobic bacteria, both as a preventive treatment (effect on planktonic growth, bacterial adhesion, and biofilm formation) and against internalization of *C. acnes* by osteoblasts, revealing that these peptides are promising as novel therapeutic candidates to prevent or treat *C. acnes* PJIs.

## INTRODUCTION

Fighting bone prosthesis infections is becoming a major public health challenge caused by the increase in surgeries involving the placement of an orthopedic implant (1). Despite antibiotic prophylaxis, usually given to patients between 30 and 60 minutes before surgery, the risk of prosthetic joint infections (PJI) is still significant (2–4). The treatment of PJI includes the removal of the infected prosthesis as well as long and specific antibiotic therapy, which contributes to their overuse (5) and the emergence of antibiotic resistance.

*Cutibacterium acnes*, an anaerobic Gram-positive bacterium, is identified in approximately 10% of PJIs (6). One of the major difficulties in diagnosing *C. acnes* infections is the absence of clinical and biological symptoms (7), which contributes to delayed diagnosis of the infection but also the risk of chronic infection, due to its slow growth and its ability to form a biofilm on both biotic and abiotic surfaces (8). Indeed, in the presence of a prosthesis, bacteria can form a biofilm with a complex composition (extracellular DNA, proteins, exopolysaccharides, bacteria under different metabolic states) conferring protection against antibiotic treatments and preventing immune system recognition (9). Moreover, the lower percentage of oxygen in bone compared to other tissues could lead to a suitable environment for *C. acnes* proliferation (10). In this clinical context, the most common antibiotic used to treat *C. acnes* PJIs is rifampicin, effective in clinical studies to treat *C. acnes* prosthesis joint infections, either alone or in combination with other antibiotics (11, 12). However, it has also been shown that the effectiveness of rifampicin may be reduced over time following repeated exposure due to the emergence of *C. acnes* resistance, largely explained by mutations in the *rpoB* gene which encodes the β-subunit of the bacterial RNA polymerase (13, 14). Since 2018, clindamycin has also been recommended in the treatment of PJIs according to the Philadelphia International Consensus and the Infectious Diseases Society of America, with significant success due to the bioavailability in bone and joint infections context (15, 16). However, *C. acnes* can also acquire resistance to clindamycin, notably by the transconjugation of the *erm* gene (17, 18).

Thus, the ability to form resistant biofilms and the inevitable emergence of antibiotic resistance in *C. acnes* PJIs underscore the urgent need for new antimicrobial strategies. Host defense peptides (HDPs), which are short and positively charged polypeptides, have emerged as a promising alternative to antibiotics, and several synthetic analogs have demonstrated favorable antibiofilm activity as well as immunomodulatory properties while exhibiting low cytotoxicity toward mammalian cells (19, 20). Thus, synthetic HDPs have the potential to address the issue of PJI-associated infections. Previous studies have shown that many peptides, particularly those comprised of D-amino acids, exhibit a broad spectrum of activity on the inhibition/eradication of biofilms targeting both Gram-positive and Gram-negative bacteria grown in aerobic conditions. For example, DJK5, a D-enantiomeric peptide targeting the stringent response, led to 99.7% and 99.8% of biofilm inhibition for the Gram-negative *Pseudomonas aeruginosa* and *Klebsiella pneumoniae* at 10 µg/mL (21). In a mouse model of *Staphylococcus aureus* cutaneous abscess infection, an environment limited in nutrient and oxygen, it was shown that DJK5 was effective *in vivo* despite the physiological tolerance of this Gram-positive bacteria embedded in a biofilm-like high-density abscess (22). The synergic effect of DJK5 and antibiotic combinations against bacterial biofilms has also been observed on Gram-negative species (21). Other peptides exhibited promising properties when combined with antibiotics, such as the broad-spectrum effect of the synthetic peptide 1018 combined with antibiotics (23).

To date, few studies have examined the treatment of *C. acnes* infections using HDPs, and most of these have focused on infections of the skin. Theansungnoen et al. showed that WSKK11 and WSRR11 peptides possessed antibacterial activity against planktonic *C. acnes* and caused abnormal morphology of bacteria cells when treated with peptide (24). In another study, Woodburn et al. studied several synthetic peptides and demonstrated that RP556 was active toward antibiotic-resistant acnes vulgaris *C. acnes* strains without significant cytotoxic effects to mammalian host cells (25). This study leads to the synthesis of novel peptides which were tested on different bacterial species responsible for skin and soft tissue infections, revealing a selective effect against *C. acnes* acneic strains at low concentrations (26). To date, one study has examined the effect of DJK5 treatment on biofilms cultivated under anaerobic conditions, specifically multispecies oral-plaque biofilms, which resulted in up to 75% killing in response to peptide treatment (27), but it is unknown whether this antibiofilm effect is specific to anaerobic environments of the oral cavity. Thus, the present study sought to determine the potential of using synthetic HDPs comprised of D-amino acids to treat biofilm-associated infections against non-oral anaerobic bacteria, such as *C. acnes*.

Our aim was to evaluate the effect of DJK5 and other synthetic D-amino acid HDPs on *C. acnes* planktonic growth and biofilm formation by determining the minimal inhibition concentration (MIC), the minimal bactericidal concentration (MBC), the minimal biofilm inhibition concentration (MBIC), as well as the impact of peptide treatment on adhered bacteria and preformed biofilm. Moreover, we evaluated the possible effect of combining these peptides with clinical antibiotics as a way to enhance their efficacy against *C. acnes* planktonic cells and prevent biofilm growth. Beyond the biofilm structure itself contributing to the failure of conventional antibiotic treatment, reduced antibiotic efficacy may also be caused by the internalization of *C. acnes* into host cells. Indeed, *C. acnes* can be internalized by host bone cells, and this phenomenon may protect the bacteria from being exposed to an effective antibiotic concentration and could increase the ability of *C. acnes* to form a biofilm (28–30). To our knowledge, no therapeutic strategies have as yet been developed against internalized *C. acne*s. Therefore, the ability of peptide treatment to prevent *C. acnes* internalization by the osteoblastic cell line, Saos-2, was also studied to determine if the peptides could prevent this bacterial protection mechanism.

## MATERIALS AND METHODS

### Bacteria strains culture

Clinical *C. acnes* strains were isolated from patients and identified by mass spectrometry at the laboratory of Reims University Hospital Center (CHU Reims). Two clinical isolates that had not induced infection were named non-PJI-related strains C2 and C5. Two others were isolated from prosthetic joint infections, PJI2 and PJI8, and were classified as PJI-related strains. *C. acnes* PJI-related strains were defined when at least three of the five samples from the bone and joint tissue during orthopedic surgery were positive after mass spectrometry analysis. Multi-locus sequence typing (MLST) profiles of the *C. acnes* strains were obtained after sequencing by Genewiz, Inc. (Leipzig, Germany) and analyzed using the online site PubMLST (https://pubmlst.org/bigsdb?db=pubmlst_pacnes_seqdef) (Table 1) (29).

**TABLE 1:** MLST profiles of *C. acnes* strains

Clinical strain	Origin	Sequence typing	Clonal complex MLST	Phylotype
C2	Skin contamination	107	CC107	IC
C5	Skin contamination	1	CC1	IA1
PJI2	Shoulder prosthesis	1	CC1	IA1
PJI8	Shoulder prosthesis	152	CC5	IB

*C. acnes* strains were isolated on Columbia agar containing 5% sheep blood (BioRad, Hercules, California, USA) and cultivated in Brain Heart Infusion (BHI) broth (BioRad, Hercules, California, USA) for 5 days, under anaerobic conditions using the GenBox system (Biomérieux, Marcy l’Etoile, France), at 37°C. For all assays, one colony of isolated *C. acnes* strains was inoculated in a 1.5 mL vial containing 1.5 mL of BHI. After 5–6 days of incubation (stationary phase reached after 4 days) at 37°C, the absorbance (at 600 nm) of the bacterial culture was adjusted to 1. The bacterial suspension was then diluted in BHI broth at 1/100 (OD: 0.01) and 1 mL of this suspension was inoculated in each well of 24- or 48-well plates.

### Antimicrobial molecules

A preliminary screen of peptides comprised of D-amino acids, such as DJK5 having excellent antibiofilm activity against Gram-negative and Gram-positive pathogens, was performed to identify peptides with promising antibiofilm activity against *C. acnes* (Table 2). D-amino acid peptides were chosen for their ability to be resistant to proteolytic degradation (31), improving the chances that these peptides would retain their activity if developed as future drug molecules (32). DJK5 and two DJK5-derivatives named AB008-D and AB009-D in which a D-Arg residue was substituted in both peptides at position five while a D-Ile or a D-Leu was substituted at position seven for AB008-D and AB009-D, respectively (see peptide sequences in Table 2). An additional peptide, AB101-D, consisting of the first eight N-terminal residues of a D-amino acid peptide, D-1002 (whose L-form has been extensively studied as an immunomodulatory peptide (33–36), joined with the last four C-terminal residues of DJK-5, was also included as part of the screen. Peptides AB008-D and AB009-D were DJK5 single amino acid substitutions, obtained from a series of 96 single amino acid substitutions of DJK5 created on peptide arrays, with moderately improved activity against *S. aureus* and *P. aeruginosa* (manuscript in preparation). A well-characterized L-amino acid peptide, 1018, with documented antibiofilm activity (37), was also included as part of the peptide screen. DJK5 was synthesized commercially by Genscript (Piscataway, NJ) while all the other peptides were synthesized by CPC Scientific (Sunnyvale, CA). All peptides were synthesized using standard solid-phase 9-fluorenylmethoxycarbonyl chemistry and purified to >95% using reverse-phase high-performance liquid chromatography. Peptide identity was confirmed by mass spectrometry. Peptides were dissolved in distilled water to a stock concentration of 1,280 µg/mL and stored at −20°C.

**TABLE 2:** Primary amino acid sequence of peptides used in this study^*a*^

Peptide	Sequence
DJK5	vqwrairvrvir-NH_2_
AB008-D	vqwr**r**i**i**vrvir-NH_2_
AB009-D	vqwr**r**i**l**vrvir-NH_2_
AB101-D	**krirwvil**rvir-NH_2_
1018	VRLIVAVRIWRR-NH_2_

^*a*^ Peptide sequences comprised of L enantiomers of amino acids are shown in upper case while D enantiomers are denoted as lower-case characters. Peptide sequence differences among the D-amino acid peptides compared to DJK5 are highlighted in bold. All peptides are amidated at their C-terminus.

Two clinical injectable solutions of antibiotics used to treat *C. acnes* infections were used: clindamycin phosphate (DALACIN C, Pfizer, Paris, France) and rifampicin (Mylan, Paris, France). Clindamycin was ready to use, at a stock concentration of 150 mg/mL and Rifampicin was dissolved with 10 mL sterile water to a final stock concentration of 60 mg/mL.

### Antimicrobial activity assays

Peptides were added to a 48-well plate at the desired concentrations in BHI medium and diluted bacteria were inoculated as described in the “Bacterial Strain Culture” section. Plates were incubated at 37°C, under anaerobic conditions using the GenBox system, for 5–6 days. The effect of the peptide on planktonic growth was determined by measuring the MIC which was defined as the concentration in the well containing the minimal amount of compound that leads to the absence of visible bacterial growth, according to Wiegand et al. (38). To determine the peptides’ MBC, 20 µL was collected from each culture supernatant containing concentrations equal to or superior to the MIC and deposited on blood agar plates. The MBC was defined as the lowest concentration of peptide without colonies after 5–6 days of incubation at 37°C, under an anaerobic atmosphere. Finally, 48-well plates containing cultures with or without peptides were washed three times with water to remove unattached bacteria. Adhered bacteria were stained with 1 mL of 0.2% crystal violet for 20 min, then washed with water, and crystal violet was extracted with 95% ethanol (vol/vol). The quantity of biofilm (biomass) was analyzed by measuring the absorbance of the crystal violet suspension at 595 nm using the SpectroStar nano spectrophotometer (BMG Labtech, Ortenberg, Germany). The Minimal Biofilm Inhibition Concentration (MBIC) was defined as the concentration in the well containing the minimal quantity of peptide where the crystal violet absorbance, corrected by the blank, was null. Experiments were performed three independent times in technical duplicate for each strain.

### Bacteria adhesion

Bacteria were inoculated in BHI in the presence of different peptide concentrations in 24-well plates containing either a Thermanox coverslip or a titanium disk. After 6 days of incubation at 37°C under anaerobic conditions, each coverslip or disk support was washed with BHI and transferred to a sterile 15 mL conical tube containing 2 mL of BHI. Adherent and biofilm-embedded bacteria were then detached using an ultrasonic bath (40 kHz) for 5 min and vortexing. A volume of 100 µL from serial dilutions was plated on blood agar plates before (non-adherent bacteria) and after (live adherent and planktonic bacteria) ultrasonic bath treatment using an EasySpiral (Interscience, Saint-Nom-la-Bretèche, France) automatic plater in exponential mode. After 5 days of incubation under an anaerobic atmosphere, the number of recovered colony-forming units (CFU) was determined using a SCAN 1200 automatic counter (Interscience, Saint-Nom-la-Bretèche, France). The quantity of live adherent bacteria was determined as follows: CFU/mL (after ultrasonic bath) – CFU/mL (before ultrasonic bath). Experiments were performed at least three independent times with technical duplicates for each *C. acnes* strain.

### Eradication assay

For eradication assays, bacteria were seeded as described in the adhesion assay above. After 5 days of incubation at 37°C under anaerobic conditions, the supernatant was removed, and the coverslip was washed twice with phosphate-buffered saline (PBS, Thermo Fisher Scientific, Waltham, Massachusetts, USA) and then transferred to a new 24-well plate. One milliliter of BHI with or without peptide at two concentrations (equivalent to the MIC or MBC) was added to the coverslip containing bacterial biofilm, and the plates were incubated for an additional 16 hours at 37°C under anaerobic conditions. Coverslips were gently washed twice with PBS, and the bacteria adherence protocol described above was used to enumerate recovered CFUs. Each experiment was performed three independent times with technical duplicates for each *C. acnes* strain.

### Scanning electronic microscopy

After 5–6 days of incubation with peptides at 16 and 32 µg/mL, coverslips were washed twice in PBS, then fixed in 1% (wt/vol) glutaraldehyde (Sigma-Aldrich, Saint-Louis, Missouri, USA) at room temperature for 1 hour. After two distilled water rinses, adherent bacteria were dehydrated in graded ethanol solutions (50, 70, 90, and twice at 100%) for 10 minutes. Finally, samples were desiccated in a drop of hexamethyldisilazane (Sigma-Aldrich, Saint-Louis, Missouri, USA). Samples were sputtered with thin gold-palladium films using a JEOL ion sputter JFC 1100 instrument (Akishima, Tokyo, Japan). Adherent bacteria were observed using a Schottky Field Emission Scanning Electron Microscope (JEOL JSM-7900F, JEOL, Akishima, Tokyo, Japan). Images were obtained at a primary beam energy of 2 kV. SEM experiments were performed two independent times, and representative images are shown in the final analysis. The length of each bacterium was measured using ImageJ software (v1.53e) (39) with a conversion between pixel and micrometer.

### Effect of peptides on osteoblast cells

#### Bacterial internalization

Bacterial internalization into osteoblast cells was determined based on a protocol adapted from Josse et al. (40). Briefly, Saos-2 osteoblast cells (ATCC, HTB-85) were seeded in 24-well plates at 10^4^ cells/cm² in culture medium and incubated at 37°C in a 5% CO_2_ humidified atmosphere for 72 hours. Saos-2 culture medium was composed of Dulbecco’s Modified Eagle Medium (DMEM) supplemented with 10% fetal bovine serum and 1% penicillin-streptomycin (PS), all of which were obtained from Thermo Fisher Scientific (Waltham MA, USA). Saos-2 cells were then washed with PBS and incubated overnight with a culture medium without PS. The following day, cells were again washed with PBS, and 1 mL of culture medium (without PS) was added with or without peptide at a concentration equivalent to MIC. One well was used for counting the number of osteoblasts per well. Concurrently, bacteria were grown as described above and then centrifuged for 5 minutes at 5,000 × *g*, and the pellets were rinsed twice with PBS and resuspended in 1 mL of PBS. Then, 10 µL of the bacterial suspension was added to the Saos-2 cell cultures to reach a theoretical multiplicity of infection (MOI) of 100:1. An initial count was performed to control the quantity of inoculum. After 3 hours of interaction, at 37°C, 5% CO_2_, supernatants were collected, and 100 µL of various dilutions was seeded on blood agar plates using the EasySpiral automatic plater. Cells were washed with PBS and incubated with cell medium containing 100 µg/mL gentamicin (Fisher Scientific, Hampton, New Hampshire, USA), for 1 hour, at 37°C, in a 5% CO_2_ humidified atmosphere to kill any bacteria that had not been internalized. Cells were again washed with PBS, and 1 mL of 0.1% Triton X-100 solution was added to each well to lyse the cells and harvest intracellular bacteria. Lysate was seeded on blood agar plates using an EasySpiral automatic seeder (Interscience, Saint-Nom-la-Bretèche, France) and incubated at 37°C under anaerobic conditions for 5 days. The number of CFU was determined using an automatic counter SCAN 1200 (Interscience, Saint-Nom-la-Bretèche, France), and the percentage of internalized bacteria was defined as follows: % of internalized bacteria = (CFU/mL × 100)/(number of osteoblasts × MOI). A control consisting of bacteria without Saos-2 cells in the presence of peptide was also prepared using the same procedure described above. Experiments were performed three independent times with technical duplicates for each *C. acnes* strain.

#### Peptide cytotoxicity

To determine the impact of peptides on osteoblasts, the lactate dehydrogenase (LDH) activity was evaluated with the cytotoxicity detection kit (Roche, Bâle, Switzerland) following the manufacturer’s instructions. Briefly, supernatants were collected after 3 hours of interaction between peptide and Saos-2 cells, filtered with a 0.22 µm filter to remove cell debris and bacteria, and then incubated with the colorimetric dye substrate for 15 minutes in the dark. Then, the absorbance was measured at 490 nm with a correction at 700 nm. Data obtained were normalized by the control, untreated cells, to observe the release of LDH. A positive control of LDH release was realized by lysing untreated cells with 1 mL of 1% Triton X-100.

### Synergy test

MIC, MBC, and MBIC were determined for clindamycin and rifampicin under the experimental conditions described above for all *C. acnes* strains.

To determine synergy, additive, or neutral effects between peptide and antibiotic, each peptide was added at different concentrations (2, 4, 8, and 16 µg/mL for each peptide) in 48-well plates. For each peptide, an antibiotic was added at different concentrations (2, 4, and 8 µg/mL for clindamycin and 1, 2, and 4 ng/mL for rifampicin). Bacteria were then inoculated in each well as previously described. After 5 days of incubation under anaerobic conditions at 37°C, the growth inhibition was recorded, and the effect of the combination was defined by calculating the fractional inhibitory concentration (FIC) as follows: FIC = (MIC_PC_/ MIC_P_) + (MIC_AC_ / MIC_A_) with _P_ for peptide, _A_ for antibiotic and _C_ for combination (Table 3) (41).

**TABLE 3:** Interpretation of FIC calculation

FIC	Effect
< 0.5	Synergist
0.5–1	Additive
1–4	Neutral
> 4	Antagonist

Synergy experiments were performed twice with two different cultures of *C. acnes*. Selected conditions where synergy was observed were performed two additional times to confirm the effect.

AB009-D peptide at 8 µg/mL and rifampicin antibiotic at 4 ng/mL were then deposited alone or in combination in 24-well plates containing Thermanox coverslip. For each condition, bacteria were inoculated to a final dilution of 1/100 from an adjusted culture with an absorbance equal to 1, in BHI. After 5 days of incubation under anaerobic conditions at 37°C, the quantity of planktonic bacteria and adhered bacteria were enumerated on blood agar plates. Experiments were performed at least three times.

### Graphical representation and statistical analysis

Results are represented as histograms with the average values and error bars representing the standard deviation of the mean. Statistical significance was assessed using the exact two-tailed non-parametric Wilcoxon-Mann-Whitney test using the GraphPad Prism (v8.0.1) software. Differences were considered significant at *P* < 0.05.

## RESULTS

### Synthetic D-amino acid HDPs impact *C. acnes* growth

The MIC and MBC of five synthetic host defense peptides were determined against four *C. acnes* strains. Three of the peptides, DJK5, AB009-D, and AB101-D had similar effects against planktonic bacteria with comparable MICs (16–32 µg/mL) and MBCs (approximately two times MIC) observed for these three peptides against the four tested strains (Table 4). The other two peptides, AB008-D and 1018, were largely inactive under these conditions with MICs and MBCs > 64 ug/mL. The impact of peptides on *C. acnes* biofilm formation was then evaluated. Interestingly, all of the peptides exerted biofilm inhibition activity (MBIC) at concentrations similar to or below those observed for their MIC, suggesting a biofilm-specific effect of the peptides on bacteria growing within a biofilm. However, DJK5, AB009-D, and AB101-D proved to be more active in this regard compared to AB008-D and 1018. Compared to conventional antibiotics used in the clinic (Table S1), the peptides had an effect at concentrations close to those of clindamycin. Rifampicin acted on the growth of *C. acnes* at a much lower concentration, and no bactericidal effect was observed under the tested conditions, except for one of the PJI-associated strains, PJI8 (Table S1). Based on their favorable antibacterial and biofilm inhibitory activities, three D-amino acid HDPs, namely DJK5, AB009-D, and AB101-D, were selected for further testing.

**TABLE 4:** MIC, MBC, and MBIC (µg/mL) of synthetic HDPs on *C. acnes* strains (C2, C5, BPI2, and BPI8) (*n* = 3 for DJK5, AB009-D, and AB101-D and *n* = 2 for 1018, AB008-D)

	DJK5			AB009-D			AB101-D			1018			AB008-D		
	MIC	MBC	MBIC	MIC	MBC	MBIC	MIC	MBC	MBIC	MIC	MBC	MBIC	MIC	MBC	MBIC
C2	16–32	32–64	16	16	32–64	8–16	16	32–64	16	>64	>64	32	32	>64	32
C5	32	32–64	32	16	32–64	8–16	16–32	32–64	16	>64	>64	32	>64	>64	32
PJI2	32	32–64	16	16	32–64	8	16–32	64	8–16	>64	>64	32	>64	>64	32
PJI8	32	64	64	16–32	64	32	16–32	64	32	>64	>64	32	>64	>64	32

### D-amino acid HDPs inhibit *C. acnes* adhesion

**Fig 1:**
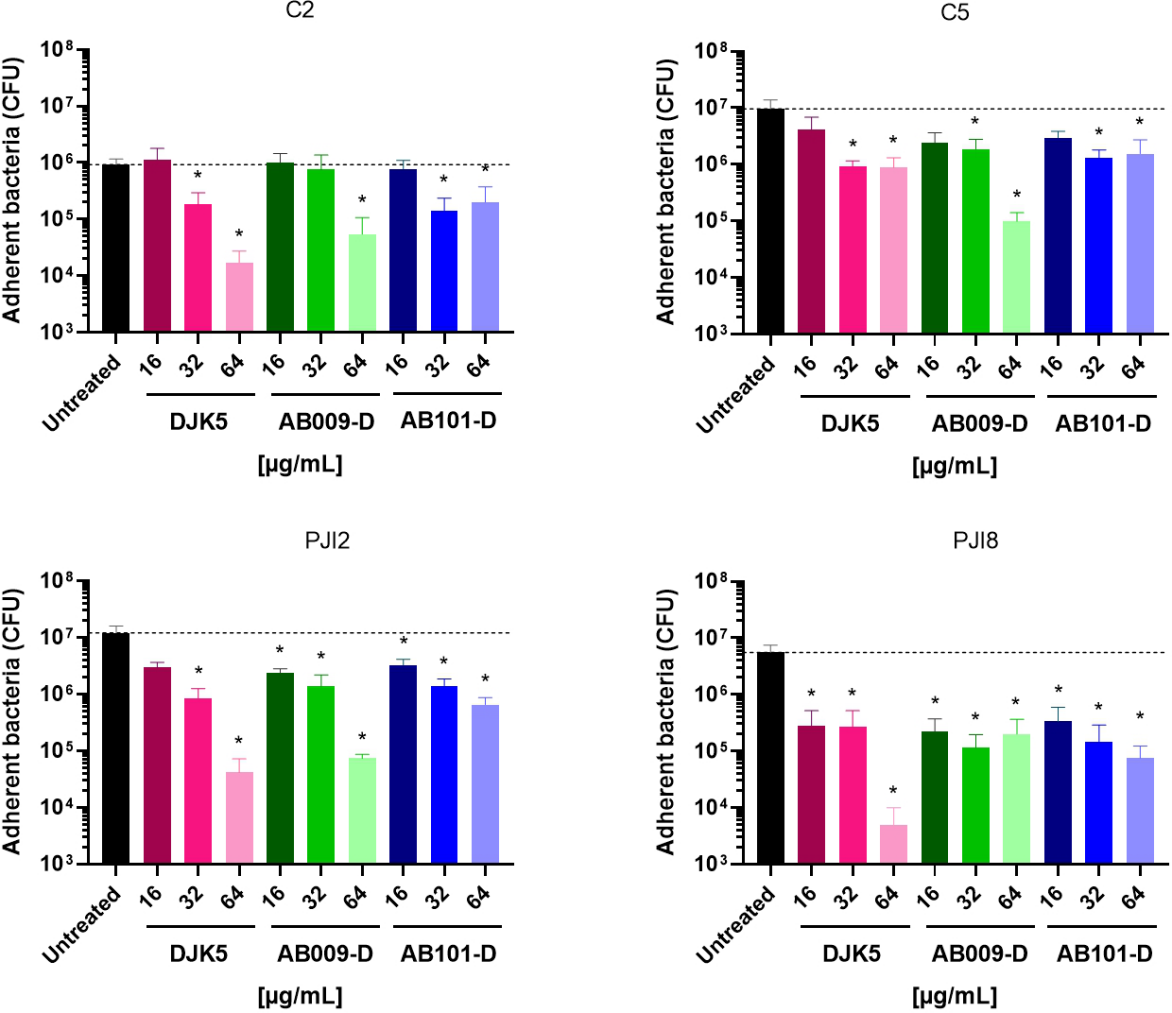
Impact of D-amino acid HDPs on *C. acnes* adhesion on plastic. Number of adherent bacteria on the surface of plastic support in the presence of different concentrations of peptides. The dotted line represents the average number of adhered cells from untreated samples. Wilcoxon-Mann-Whitney test: versus untreated bacteria *P < 0.05, at least three independent experiments with two technical replicates (representation of the average of each condition ± SD).

To further examine the impact of the synthetic D-amino acid peptides on *C. acnes* biofilm growth, the effect of the three most active peptides on bacterial adhesion was investigated by counting the quantity of adherent and live bacteria on the surface of plastic supports after 5 days of treatment. Overall, peptide treatment resulted in a decrease in the number of bacteria adhered on plastic supports in a dose-dependent manner, with DJK5 exhibiting the strongest anti-adhesion properties for all strains with a significant 5-fold to 16.5-fold decrease at 32 µg/mL (Fig. 1). At 32 µg/mL, a decrease in the number of adhered bacteria was noticed for the PJI-related *C. acnes* strains with AB009-D (significant, 5.1- and 27.1-fold reduction for PJI2 and PJI8, respectively) and AB101-D (significant, 3.7-fold and 11-fold for PJI2 and PJI8, respectively) compared to untreated samples (Fig. 1). At 64 µg/mL, a notable decrease in bacterial adhesion was observed for both PJI-related strains and C2 when treated with DJK5 compared to the other two peptides at the same concentration. Globally, a significant 2-log decrease in bacterial adhesion on plastic substrate was observed for every *C. acnes* strain, with DJK5 and/or AB009-D having the strongest effect when tested at their MBC concentration of 64 μg/mL (Fig. 1).

**Fig 2:**
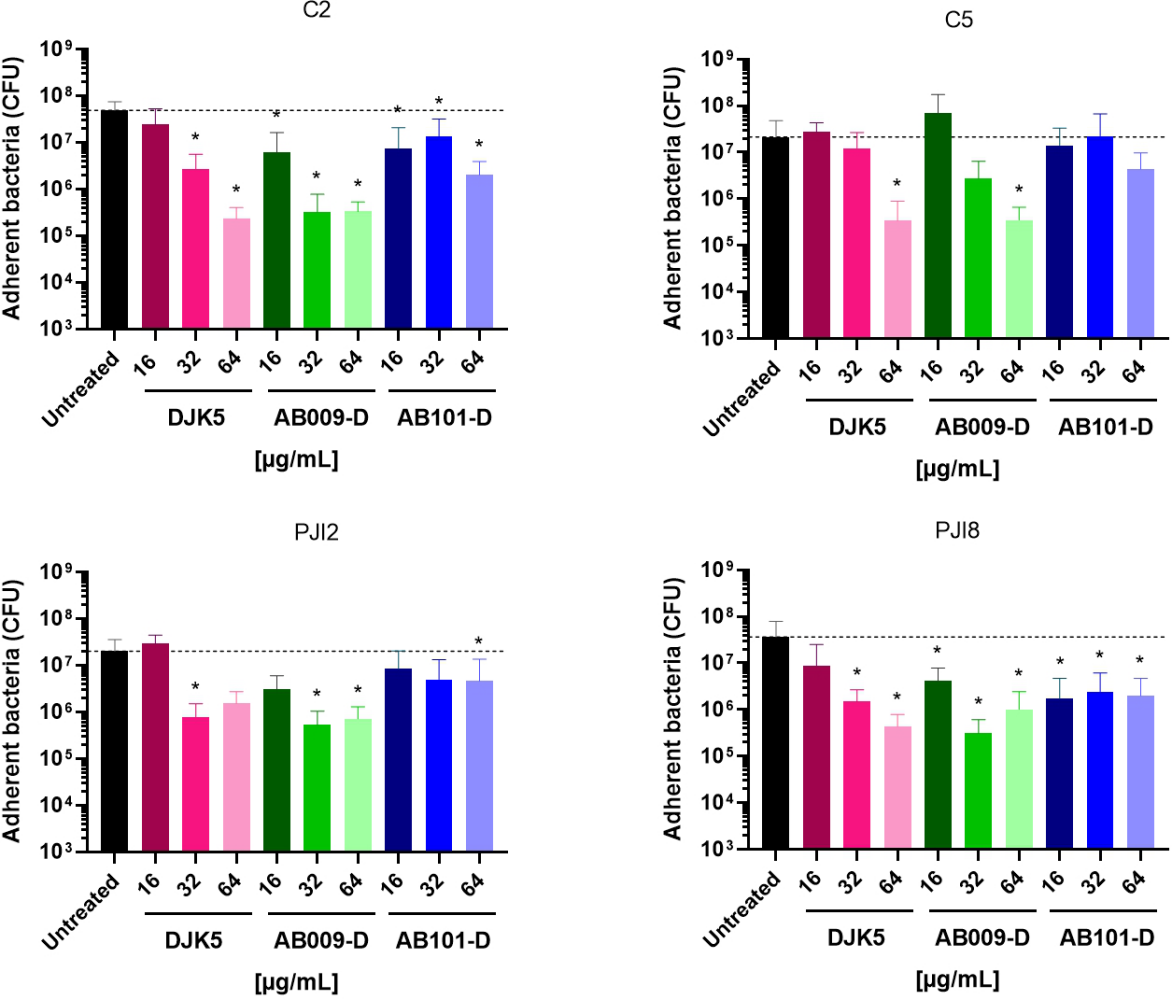
Effect of D-amino acid HDPs on *C. acnes* adhesion on titanium. Quantity of adherent bacteria on titanium in the presence of peptides. The dotted line represents untreated bacteria. Wilcoxon-Mann-Whitney test: versus untreated bacteria *P < 0.05. *n* = 3 to 5 independent experiments (representation of the average of each condition ± SD).

The adhesion of *C. acnes* on titanium supports, which is the material commonly used for bone prosthetic manufacturing, was also quantified when treated with peptide. All three peptides reduced the ability of nearly all *C. acnes* strains to adhere on titanium supports in a largely dose-dependent manner (Fig. 2). The response to AB009-D treatment was similar to that seen for DJK5, with a significant decrease in adhered cells in the presence of 32 µg/mL peptide and an ~2 log decrease in adhered cells at the highest concentration of peptide tested of 64 µg/mL. The anti-adhesion activity of AB101-D on *C. acnes* adhesion to titanium was not as pronounced and appeared to be attenuated against the C5 strain.

### D-amino acid HDPs impact *C. acnes* morphology

**Fig 3:**
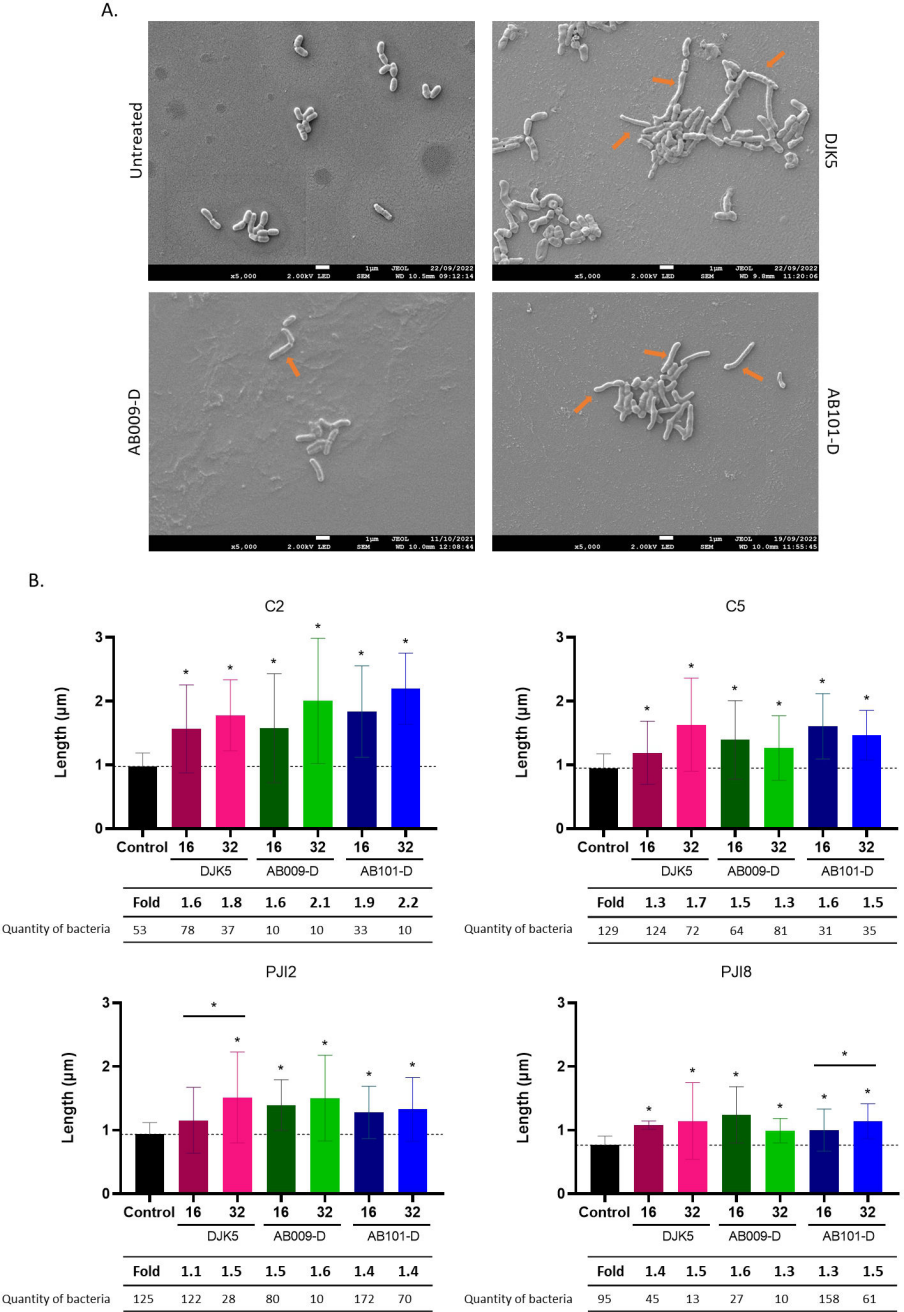
Impact of D-amino acid HDPs treatment on *C. acnes* cell length. (**A**) Observation of *C. acnes* C2 cell morphology treated with synthetic D-amino acid peptides by scanning electron microscopy. Images were obtained at 5,000× magnification with a scale of 1 µm (blank line) shown for each condition. Orange arrows highlight elongated bacteria. (**B**) Average length (±SD) of each *C. acnes* bacteria strain in the presence of peptide, as determined using ImageJ software. At least 10 bacteria were counted for each condition, according to the concentration of peptides. Wilcoxon-Mann-Whitney test: versus untreated bacteria *P < 0.05. *n* = 2 independent experiments.

The effect of the D-amino acid HDPs on *C. acnes* cell morphology was examined by scanning electron microscopy (Fig. 3A). It was observed that 5 days of peptide treatment impacted *C. acnes* morphology with a significant increase in bacterial cell length observed with this effect being particularly notable for the C2 strain (Fig. 3B).

Overall, the synthetic D-amino acid HDPs were able to kill planktonic bacteria and prevented *C. acnes* adhesion while also modifying *C. acnes* cell morphology in the early stages of biofilm development. To further examine the effect of the peptides on *C. acnes* biofilms that may already be present in PJIs, it was necessary to evaluate if any of the peptides could eradicate pre-formed and/or mature biofilms of *C. acnes*.

### D-amino acid HDPs reduce viability of mature biofilm

**Fig 4:**
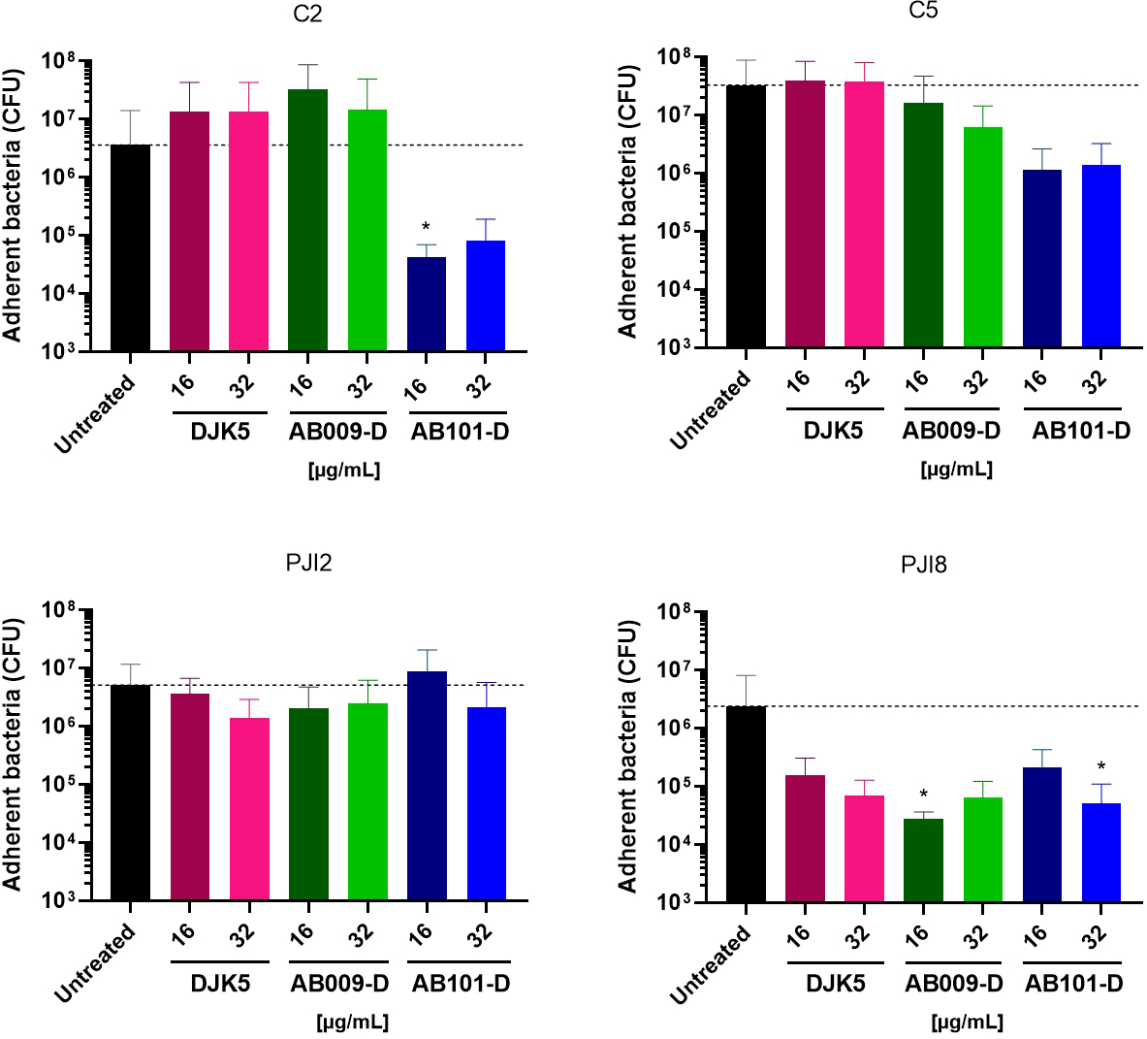
Impact of overnight D-amino acid HDP treatment on *C. acnes*-formed biofilm. Adherent bacteria in formed biofilm after 16 hours of D-amino Acid HDPs treatment normalized versus untreated biofilm Wilcoxon-Mann-Whitney test: versus untreated bacteria *P < 0.05. *n* = 4 independent experiments with one or two technical replicates (representation of the average of each condition ± SD).

Near MIC concentrations (16 µg/mL and 32 µg/mL) of D-amino acid HDPs were added to a 5-day-old mature biofilm for 16 hours. Compared to untreated biofilm, a decrease in the quantity of *C. acnes* bacteria was observed for three of the four strains treated with AB101-D, with notable differences according to strain (100.1-fold, 28.2-fold, and 11.6-fold decrease at 16 µg/mL compared to untreated for C2, C5, and PJI8, respectively) (Fig. 4). Interestingly, DJK5 and AB009-D treatment did not substantially reduce the number of *C. acnes* cells in 5-day-old biofilms except for the PJI8 strain, which appeared to be sensitive to all of the peptides under these conditions. Conversely, biofilms formed by strain PJI2 appeared to resist the effects of all the peptides under these conditions.

Overall, AB101-D exhibited the most potent antibiofilm activity against pre-formed *C. acnes* biofilms as it was able to reduce the number of viable bacterial cells recovered from mature biofilm, with a strain-dependent response.

### Effect of D-amino acid HDPs on *C. acnes* internalization in osteoblasts

To evaluate the effect of the peptide on *C. acnes* internalization by osteoblast cells, peptides at concentrations equal to the MIC (32 µg/mL for DJK5 and 16 µg/mL for AB009-D and AB101-D) were added during the 3 hours of co-culture with each *C. acnes* strain. The amount of released LDH in the culture supernatant was then assessed using a cytotoxicity assay kit. No enhanced cytotoxicity of peptides on osteoblasts compared to untreated cells was observed (Fig. S1) suggesting that the peptides themselves were not causing cell lysis that may confound the results of the bacterial internalization assay.

**Fig 5:**
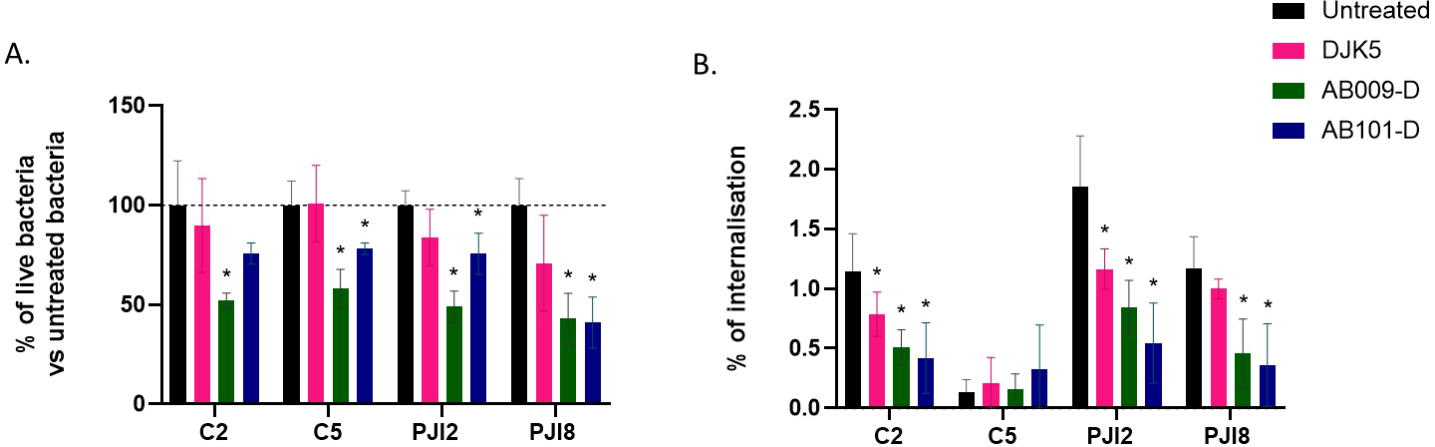
Impact of D-amino acid HDPs at 16 µg/mL or 32 µg/mL on *C. acnes* internalization by Saos-2 cell line. (**A**) Percentage of *C. acnes* viability in the medium after 3 h of incubation with D-amino Acid HDPs. (**B**) Percentage of *C. acnes* internalization by Saos-2 cells. The dotted line represents the percentage of untreated conditions. Wilcoxon-Mann-Whitney test: versus untreated bacteria *P < 0.05. *n* = 3 independent experiments with two technical replicates (representation of the average of each condition ± SD).

In the presence of peptide and added *C. acnes* cells, a significant decrease in extracellular bacteria was observed in the presence of AB009-D (41.9%–57.1% reduction across all four *C. acnes* strains) and AB101-D (21.9%–59% reduction) compared to untreated bacteria (Fig. 5B). Interestingly, no significant effect of DJK5 was noted on planktonic *C. acnes*.

The percentage of internalization of three *C. acnes* strains, namely C2, PJI2, and PJI8, significantly decreased in the presence of the D-amino acid HDPs (Fig. 5C). However, it should be noted that this effect could be a consequence of the rapid bactericidal impact of AB009-D and AB101-D on extracellular *C. acnes* rather than either peptide preventing bacterial internalization. Interestingly, in the case of DJK5, an inhibition of internalization of C2 and PJI2 by osteoblasts (0.79% vs 1.15% for C2 and 1.16% vs 1.85% for PJI2) was observed without impacting *C. acnes* planktonic growth.

### Combination of antibiotic and D-amino acid synthetic HDP treatment

**Fig 6:**
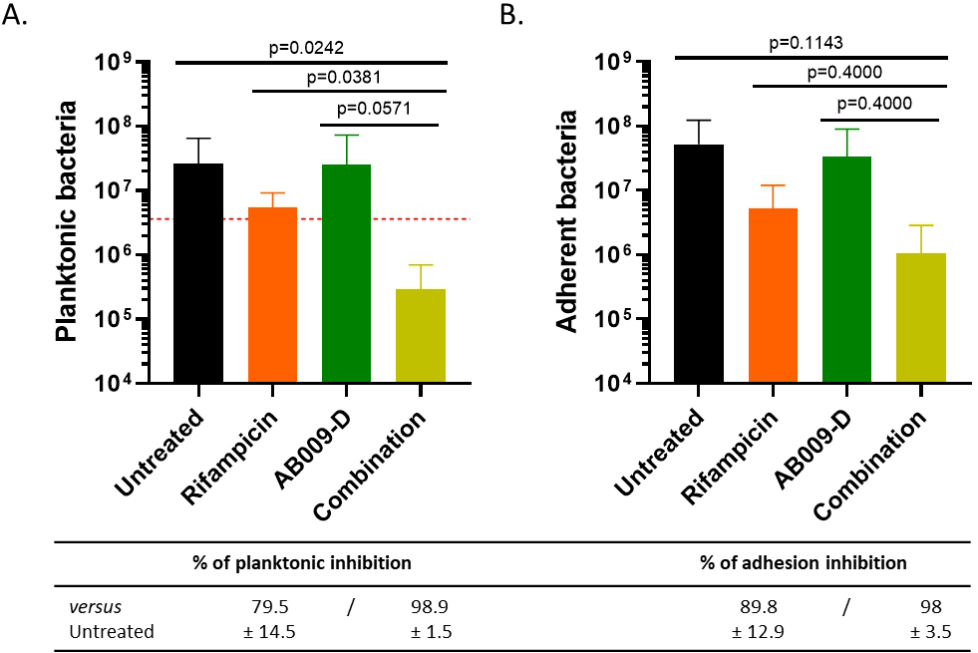
Impact of AB009-D at 8 µg/mL (twofold drop MIC) with rifampicin at 4 ng/mL (twofold drop MIC) on C2 *C. acnes* strain. (**A**) Quantity of planktonic bacteria and (**B**) adherent bacteria on plastic coverslip after 5 days of AB009-D and rifampicin treatment alone or in combination. The red dotted line represents the number of bacteria inoculated. *n* = 2 independent experiments with two technical replicates (representation of the average of each condition ± SD).

In the perspective of a dual strategy with other antimicrobial molecules, the effect of combined therapy with conventional antibiotics used to treat *C. acnes* infection was studied. For that, rifampicin or clindamycin was added at sub-MIC concentration along a range of concentration peptides, and the FIC was calculated to quantify the effect of antibiotic and peptide combination on *C. acnes* planktonic growth. Results showed a neutral effect for most conditions, except for C2 with a slight additive effect in the presence of 4 ng/mL of rifampicin and 16 µg/mL of AB009-D on *C. acnes* growth (2–4 times fold MIC drop) (Table 5). To further characterize this additive effect, this latter result was confirmed by CFU enumeration showing a decrease in bacterial concentration in both planktonic phases (94.6% ± 7.5% of planktonic inhibition compared to rifampicin alone and 98.8% ± 1.6% of planktonic inhibition compared to AB009-D alone) and adherent phase (80.1% ± 34% of adhesion inhibition compared to rifampicin alone and 96.9% ± 5.4% of adhesion inhibition compared to AB009-D alone) (Fig. 6).

**TABLE 5:** Effect of D-amino acid HDPs added with rifampicin fixed to 4 ng/mL or clindamycin fixed to 8 µg/mL (twofold drop of antibiotics MICs) on *C. acnes^a^*

Strain	DJK5			AB009-D			AB101-D		
	MIC_combined_	Fold drop	FIC	MIC_combined_	Fold drop	FIC	MIC_combined_	Fold drop	FIC
Rifampicin 4 ng/mL									
C2	8–16	1–2	1–1.5	4–8	2–4	**0.75–1**	8–16	1–2	1–1.5
C5	16–32	1–2	1–1.5	8	2	**1**	16	1	1.5
PJI2	16	2	**1**	8–16	1–2	1–1.5	8–16	1–2	1–1.5
PJI8	16	2	**1**	8–16	1–2	1–1.5	8–16	1–2	1–1.5
Clindamycin 8 µg/mL									
C2	32	1	1.5	8–16	1–2	1–1.5	16	1	1.5
C5	32	1	1.25	16	1	1.25	16	1	1.25
PJI2	32	1	1.5	16 - > 16	/^*b*^	/	16	1	1.5
PJI8	32	1	1.25	16 - > 16	/	/	16 - > 16	/	/

^*a*^ Determination of minimal planktonic inhibition concentration (MIC_combined_, µg/mL) and fractional inhibitory concentration (FIC). The additive effect is shown in bold. n=2 independent experiments with 2 different cultures of *C. acnes*.

^*b*^ "/", no value.

## DISCUSSION

*C. acnes* PJIs are complicated and frequently lead to surgical treatment (15) but also often require a heavy and long antibiotic therapy regimen that may contribute to the emergence of bacteria that are tolerant or even resistant to the effects of antibiotics (5). Beyond the emergence of antibiotic resistance, *C. acnes* infection persistence can be partly explained by biofilm formation, which confers a level of adaptive resistance to the effects of antibiotics (8). To fight *C. acnes* biofilm-associated infections, three antimicrobial strategies are envisaged: prevention of biofilm attachment, the killing of bacterial cells within a biofilm, and/or dispersion of the bacterial cells from the biofilm structure (42). *C. acnes* can also be internalized by bone cells, permitting the bacterial cells to evade the immune system and leading to a modification of its behavior that enhances its capacity to form a biofilm (29, 30). To avoid *C. acnes* internalization, two strategies could be considered: killing planktonic or adherent bacteria and/or blocking bacteria internalization by host cells, the latter being a strategy not widely explored in the literature. Thus, to combat *C. acnes* PJIs, novel approaches using new chemical entities are needed that possess antimicrobial, antibiofilm, and/or anti-internalization activities.

Effective broad-spectrum antibiofilm and host defense peptides represent a potential preventive action and/or treatment option for *C. acnes* PJIs. However, their effect on anaerobic bacteria, and specifically on *C. acnes,* has not been widely studied, and this needs to be appreciated due to the prevalence and distribution of this type of organism in PJIs (43). To address this knowledge gap, the present study examined the effect of synthetic HDPs on *C. acnes* planktonic growth as well as characterized their impact on both biofilm initiation and eradication, and also on the internalization of *C. acnes* by osteoblasts.

DJK5 peptide was used as a reference peptide, already determined as a promising antibiofilm molecule against broad-spectrum species but never tested against anaerobe *C. acnes* and for its anti-internalization effect. Three synthetic D-amino acid peptides (DJK5, AB009-D, and AB101-D) were identified that exhibited a bactericidal effect on *C. acnes* with rapid action for AB009-D and AB101-D (around 50% of a decrease in planktonic bacteria after 3 hours of incubation). DJK5 inhibited both biofilm initiation and internalization into Saos-2 cells. AB009-D proved especially effective at preventing biofilm initiation, and AB101-D was particularly effective against pre-formed biofilm. These results suggest that differences in the primary amino acid sequence of a peptide can shift the activity profile of highly similar peptide sequences. For instance, AB009-D differs from DJK5 at only two positions, a D-Arg residue at position 5 and a D-Leu residue at position 7, and largely retains the activity of the parent sequence. However, a similarly modified peptide sequence, AB008-D, in which the D-Arg substitution at position 5 is preserved but a D-Ile residue is instead substituted at position 7, was comparatively inactive against *C. acnes*. Moreover, the difference in response to peptide treatment of the two clinical strains (C5 and PJI2), having the same MLST profile, shows that the identification of the MLST profile does not inform on *C. acnes* response to peptide treatment.

In a previous study on Gram-negative bacteria grown under aerobic conditions, biofilm inhibition concentration of peptides comprised of D-enantiomeric amino acids was lower than their MIC, from 2-fold up to 16-fold less (21). In our hands, we similarly observed that D-enantiomeric HDPs were efficient against anaerobic *C. acnes* species with an MBIC equal to or lower than their MIC. The selective activity of these peptides could be linked to the metabolic differences between planktonic bacteria and bacteria growing as biofilms (37). DJK5 and AB009-D peptides inhibited biofilm formation on both plastic and titanium supports for all *C. acnes* strains with a 2-log decrease in adherent *C. acnes* in the presence of peptide at its MBC. DJK5 has previously been shown to reduce biomass of anaerobic oral biofilm (27) and biofilms grown aerobically on porous titanium (44), and our results support the antimicrobial effect of D-enantiomeric HDPs against bacteria grown under an anaerobic atmosphere and on titanium alloy surfaces.

In 2015, de la Fuente-Nuñez et al. demonstrated that DJK5 can disperse and eradicate *P. aeruginosa* biofilm using the dynamic flow cell method (21). In this study, DJK5 and AB009-D also acted on mature biofilm, albeit in a static microtiter plate biofilm growth model, but only for one *C. acnes* strain with an observed decrease of live and adherent bacteria. Interestingly, for AB101-D, it seems that the peptide had a stronger effect on pre-formed biofilms, effectively reducing the number of bacterial cells recovered across three of the four strains evaluated and suggesting that this peptide more effectively killed and/or dispersed bacteria embedded within the biofilm.

In bone and joint infections, *C. acnes* interacts with the host bone environment. Indeed, *C. acnes* could escape antimicrobial treatment by being internalized into osteoblast cells (28, 29). Therefore, inhibition of internalization could reduce the persistence of *C. acnes* in PJIs. Here, treatment with DJK5 decreased the quantity of osteoblast internalized bacteria despite an absence of rapid action on external and planktonic growth, whereas the observed decrease in internalization rates in the presence of AB009-D and AB101-D appeared to be mainly due to the killing of planktonic bacteria by peptide treatment.

In our study, an elongation of *C. acnes* length in response to peptide treatment was observed by SEM which suggests an impairment of cell division caused by the peptide, with the most pronounced effect occurring with peptide treatment of the non-related-PJI strain, C2. The link between cell length and its consequences on bacterial adhesion and internalization by osteoblasts has to be explored. A similar phenomenon was observed by Di Somma et al., with the AMP Temporin L, which acted on the cell division of *Escherichia coli* by binding FtsZ (45). FtsZ is a protein found in many bacterial species, notably in MRSA strains (Gram-positive bacterial strains), which functions as a filamentous GTPase that forms the Z-ring responsible for cell division. Han et al. have listed some peptide-like molecules that affect FtsZ assembly leading to an inhibition of Z-ring formation and cell division (46). In a recent study, Jiang et al. showed that FtsZ protein was upregulated in membrane vesicles of antibiotic-resistant *C. acnes* strains from lesions of an acne patient (47). This result reveals that FtsZ is expressed in some *C. acnes* strains and may explain the mechanism underlying the impact of the D-amino acid HDPs on *C. acnes* cell growth.

The antibiofilm mechanism of action of DJK5 was previously characterized in other bacteria species, which implicated the (p)ppGpp molecule as a molecular target involved in biofilm formation and growth (21, 22, 37, 48). The gene coding for (p)ppGpp in *C. acnes* was identified in 2013 after whole-genome sequencing in HL096PA1 (49). Therefore, we can speculate that the mechanism of action of DJK5 and the other D-amino acid synthetic HDPs against *C. acnes* could be similar to other species. However, no study to date has demonstrated the role of ppGpp in biofilm formation for this species, and therefore the consequence of the interaction between D-enantiomeric HDPs and ppGpp has yet to be elucidated for *C. acnes*. Other mechanisms of action could be in play for the HDP activity seen against *C. acnes*, such as disturbance of bacterial cell wall (50), prevention of cell division (46), and blockage of initial attachment (51).

To avoid antibiotic tolerance and antibiotic resistance emergence, new strategies are being explored, like the association of different molecules with distinct mechanisms of action to avoid the selection of resistant bacteria and to decrease the antibiotic concentrations. Thus, de la Fuente-Nuñez et al. showed that DJK5 combined with commonly used antibiotics, like ciprofloxacin, had a synergistic effect on *P. aeruginosa* with a 2-fold to 16-fold decrease in antibiotic concentration. However, this effect was strain-dependent, with only an additive effect observed on other Gram-negative strains (21). Another peptide (1018) enhanced the action of antibiotics on Gram-positive and Gram-negative strains (23). Two common antibiotics (clindamycin and rifampicin) (52) used for *C. acnes* infections were tested with the synthetic D-enantiomeric HDPs evaluated in our study. The results obtained showed that the combination of AB009-D with rifampicin revealed a slight additive effect on preventing *C. acnes* biofilm growth on plastic supports for one strain. Therefore, using a peptide could enhance the effectiveness of an antibiotic treatment, thus enabling the use of lower concentrations of antibiotics which can help reduce the risk of developing antibiotic tolerance or resistance. This effect needs to be examined in other *C. acnes* strains to confirm the relevance of a combination treatment, but it also underscores the need to optimize the peptide sequence to target *C. acnes* adhesion and biofilm.

In conclusion, synthetic D-amino acid HDPs exhibited antimicrobial and antibiofilm activity against *C. acnes,* although different peptides were better at exerting particular anti-*C*. *acnes* activities than others, and certain strains of *C. acnes* were more susceptible to the effects of peptides. Overall, this study demonstrates that peptide sequences could be optimized with tailored activity profiles that prevent *C. acnes* growth under conditions that are found in PJI infections, and/or target *C. acnes* that has been internalized into cells, both of which would improve their utility as potential future treatment options for *C. acnes*-associated PJIs.

## SUPPLEMENTAL MATERIAL

Table S1 and Figure S1 spectrum.02523-24-s0001.docx Table S1: MIC, MBC and MBIC of conventional antibiotic used in clinic to treat C. acnes PJIs, n=3. Figure S1: Peptide and bacteria cytotoxicity by the LDH release assay.

ASM does not own the copyrights to Supplemental Material that may be linked to, or accessed through, an article. The authors have granted ASM a non-exclusive, world-wide license to publish the Supplemental Material files. Please contact the corresponding author directly for reuse.
