# Supplementary material for: D-enantiomeric antibiofilm peptides effective against anaerobic Cutibacterium acnes biofilm
Source: Microbiol Spectr. 2025 Mar 25;13(5):e02523-24. doi: 10.1128/spectrum.02523-24 (PMC12053997; doi:10.1128/spectrum.02523-24)
Supplement: Table S1 and Figure S1 — Table S1: MIC, MBC and MBIC of conventional antibiotic used in clinic to treat C. acnes PJIs, n=3. Figure S1: Peptide and bacteria cytotoxicity by the LDH release assay. [file spectrum.02523-24-s0001.docx]

|  |  | Clindamycin (µg/mL) | | |  | Rifampicin (ng/mL) | | |
| --- | --- | --- | --- | --- | --- | --- | --- | --- |
|  |  | MIC | MBC | MBIC |  | MIC | MBC | MBIC |
| C2 |  | 16-32 | 64 | 16 |  | 8 | >64 | 2-4 |
| C5 |  | 32 | 64 | 32 |  | 8 | >64 | 8 |
| PJI2 |  | 16-32 | 64 | 16-32 |  | 8 | >64 | 4-8 |
| PJI8 |  | 32 | 64 | 32 |  | 8 | 8-16 | 8 |

**TABLE S1** MIC, MBC and MBIC of conventional antibiotic used in clinic to treat *C. acnes* PJIs, n=3.

**FIG S1** Peptide and bacteria cytotoxicity by the LDH release assay.

n= 3 independent experiment with 2 technical replicates (representation of the average of each condition ±SD).
